# 460. SARS-CoV-2 Infection-Induced Antibody Seroprevalence in Previously Infected Persons with Immunocompromising Conditions — United States, 2020–2022

**DOI:** 10.1093/ofid/ofad500.530

**Published:** 2023-11-27

**Authors:** Anna Bratcher, Jefferson M Jones, Aaron Harris, Kristie E N Clarke

**Affiliations:** CDC - Atlanta, GA, Atlanta, Georgia; CDC, Atlanta, Georgia; Centers for Disease Control and Prevention, Atlanta, Georgia; CDC, Atlanta, Georgia

## Abstract

**Background:**

People with immunocompromising conditions (IC) are at increased risk of severe COVID-19 and death. These individuals show weaker immunogenicity following vaccination than individuals without IC, yet immunogenicity after SARS-CoV-2 infection is poorly understood. To address this gap, the presence of infection-induced antibodies in sera following a positive COVID-19 test result was compared between people with and without IC.

**Methods:**

Data were from CDC’s national commercial laboratory seroprevalence study, a repeated, cross-sectional survey that includes associated diagnostic codes and previous COVID-19 viral test results. Infection-induced antibody seroprevalence in sera from people with a positive COVID-19 test result was compared by IC status for three post-infection periods: 14–90 (early), 91–180 (mid), and >180 (late) days. A logistic regression produced adjusted odds ratios (aOR) comparing infection-induced antibody prevalence among specimens with and without associated IC adjusted for age, sex, and infection-induced antibody assay used (Abbott Architect, Ortho VITROS, or Roche Elecsys).

**Results:**

The analytic sample consisted of 15,554 specimens across the three periods (4,571 early, 4,465 mid, and 6,518 late). Of these, 188, 157, and 283 specimens had one or more associated, recorded IC, respectively. During the early period, 22.3% of specimens with IC lacked infection-induced antibodies compared with 6.8% of those without IC. After adjustment, specimens with IC were more likely to lack infection-induced antibodies in the early (aOR: 4.85; 95% CI: 3.20–7.38), mid (aOR: 2.53; 95% CI:1.57–4.09), and late (aOR:1.62; 95% CI:1.12–2.36) periods compared to specimens without IC.

**Conclusion:**

Sera from people with IC is less likely to contain infection-induced antibodies following SARS-CoV-2 infection compared to sera from those without IC within the studied periods. These findings stress the importance of prevention measures for people with IC, such as pre-exposure prophylaxis, additional vaccination doses, and consistent mask use before and after a documented infection.

**Disclosures:**

**All Authors**: No reported disclosures

